# A retrospective study of infantile-onset Takayasu arteritis: experience from a tertiary referral center in China

**DOI:** 10.3389/fcvm.2024.1249305

**Published:** 2024-01-31

**Authors:** Jing Jin, Yan Zhao, Xiucheng Gao, Panpan Wang, Yingying Liu, Yuting Pan, Zhidan Fan, Haiguo Yu

**Affiliations:** ^1^ Department of Rheumatology and Immunology, Children’s Hospital of Nanjing Medical University, Nanjing, China; ^2^ Department of Ultrasonography, Children’s Hospital of Nanjing Medical University, Nanjing, China; ^3^ Department of Image, Children’s Hospital of Nanjing Medical University, Nanjing, China

**Keywords:** Takayasu arteritis, infant, disease activity score, biologic therapy, corticosteroids

## Abstract

**Objective:**

Takayasu artery (TAK) is a chronic inflammatory disease that mainly affects the aorta and its major branches and is rarely reported in infants. We aimed to summarize the clinical features of infant TA (I-TA) in a tertiary care center.

**Methods:**

We performed a retrospective study involving 10 infants diagnosed with TAK. A comprehensive evaluation of clinical, laboratory, radiographic features, disease activity, treatment and outcomes was carried out.

**Results:**

A consecutive cohort was composed of 8 girls and 2 boys, with an age at diagnosis of 11.1 (1.7–36) months. The median time to diagnosis and the average time to follow-up were 9.5 days (2–235 days) and 10.9 (1–21) months, respectively. The most common initial manifestations were malaise (80%), fever (70%), hypertension (50%) and rash (30%). The mean Pediatric Vasculitis Activity Score (PVAS), Takayasu Clinical Activity Score (ITAS-2010) and ITAS-A scores were 2.8/63, 2.6/51, and 5.6/54, respectively. All patients had aberrant laboratory parameters. The most common lesions were in the thoracic aorta (60%) and abdominal aorta (60%). Corticosteroids combined with cyclophosphamide followed by long-term mycophenolate mofetil were initiated in most cases (70%). Biologics were attempted in 5 cases. Mortality was 40%.

**Conclusions:**

It is challenging to diagnose TAK in infants in a timely manner. Considering the more vessels involved, more severe inflammation and higher mortality, aggressive treatment is warranted in infants. GCs and CYC treatment seem to be effective.

## 1 Introduction

TAK is an idiopathic large vessel vasculitis that affects the aorta and its main branches at their origin and leads to systemic inflammation and ischemia of involved organs (1). It commonly occurs in the third and fourth decades of life but rarely in infants and children (2). Although the incidence of TAK is extremely low in infants, the occurrence of multivessel involvement, severe systemic inflammation and the delay of diagnosis lead to a poor prognosis.

Unfortunately, diagnostic delay is quite common in the clinic on account of nonspecific symptoms at the acute stage of disease and the paucity of validated biomarkers to assess its activity and damage, even years after the acute onset of TAK, causing stenosis and fibrosis of involved vessels (3). Moreover, the choice of treatment protocol remains a major challenge in TAK since there is a scarcity of high-quality evidence on which to base treatment recommendations, especially in infants with limited data. To date, there are few reports regarding the clinical profile and outcome of infant TAK.

Here, we present the clinical features and follow-up data of infants diagnosed with TAK from a single Chinese center to describe the limited infant TAK experience available to prompt the early diagnosis and prognosis of this rare vasculitis.

## 2 Methods

In this retrospective study, ten patients who met the diagnostic criteria of childhood TAK and had onset of illness during their first three years of life were enrolled at our hospital from January 2017 to February 2022. According to the 2008 EULAR/PRINTO/PRES criteria(4), patients were diagnosed with TAK with angiographic abnormality (conventional, CT, or MRI) of the aorta or its main branches and pulmonary arteries (mandatory criterion) plus at least one of the following: (1) absence of the peripheral artery pulse or claudication induced by physical activity; (2) a > 10 mm Hg difference in systolic BP in all four limbs; (3) Bruits over large arteries; (4) hypertension (when compared with age-matched healthy children); and (5) increased levels of acute phase reactants erythrocyte sedimentation rate (ESR) and/or C reactive protein (CRP). Patients who concurrently suffered from other inflammatory diseases were excluded.

This study was approved by the Ethical Committee of Children's Hospital of Nanjing Medical University (202302039-1).

Three tools were used to document disease activity both at disease onset and at the last follow-up: the Pediatric Vasculitis Activity Score (PVAS), Takayasu Clinical Activity Score (ITAS-2010) and ITAS-A. The PVAS is specific for pediatric vasculitis and is derived from the BVAS, with a total score of 63, while the ITAS-2010 is specific for TAK, with a maximum score of 51. ITAS-A is calculated by adding the score for acute phase reactant (CRP or ESR) to the total score of ITAS-2010, which increases the maximum score to 54 (5, 6).

An effective treatment was defined as improved clinical symptoms, no radiographic progression (even radiographic remission), and a reduction in the disease activity scores mentioned above.

These infants were followed up every 1 month for the first 6 months after initiation of treatment and then every 2–3 months for 4–21 months via regular clinic visits or by telephone interview.

### 2.1 Statistical analysis

To summarize patient characteristics, values are presented as the mean with standard deviation for continuous variables or frequencies with percentages for qualitative variables. Continuous variables were compared using Student's t-tests. Spearman's rank correlation analysis was used to examine associations between the PVAS and ITAS. *P*-values < 0.05 were considered significant.

## 3 Results

### 3.1 Baseline characteristics

A total of 10 infants with TAK were included in the analysis, comprising 8 girls and 2 boys. The ages of onset ranged from 1.7 months to 3 years. The median time to diagnosis was 9.5 days (range, 2–235 days). No family history of vasculitis or other inflammatory diseases was gained.

### 3.2 Clinical manifestations

In addition to malaise (80%), fever (axillary or tympanic temperature ≥38°C) was the most common clinical manifestation seen in 7 cases (70%), which occurred consistently or transiently. Additionally, five (50%) infants had hypertension (≥95th percentile), and three (30%) had rash. Other clinical features include blood pressure (BP) discrepancy (20%), weak pulse (20%), red eye conjunctivitis (20%), vomit (20%), anorexia (20%), headache (10%), abdominal pain (10%) and finger ulcer (10%). In addition, the initial presentation and disease activity scores (PVAS/ITAS-2010/ITAS-A) at the first clinic visit are shown in Table 1. In our study, ITAS-A score is a sum of ITAS score with CRP which is ITAS-A (CRP).

**Table 1:** Time to diagnosis, initial presentation, disease activity scores, outcomes and treatment.

Patient number	Gender	Age of initial presentation (Mo)	Time to diagnosis (D)	Initial presentation	PVAS/ITAS/ITAS-A score at first clinic	Follow-up (Mo)	Status at last follow-up	Treatment
1	F	2.8	18	Vomit, malaise, hypertension	2/2/5	9	R	IVIG + GC + CTX + MMF
2	F	1.7	120	Fever, rash	2/1/4	9	R	IVIG + GC + IFX + CTX + TCZ
3	F	36	4	Fever, headache, conjunctivitis, hypertension	4/2/5	21	R	GC + CTX + MMF
4	M	14	235	Fever, rash, conjunctivitis, malaise	2/4/7	1	D	IVIG + GC + IFX + COX + CTX
5	M	36	14	Fever, rash, abdominal pain, malaise	4/3/6	7	R	IVIG + GC + IFX + CTX
6	F	5.7	2	Fever, malaise, BP discrepancy	4/3/6	7	R	IVIG + GC + IFX + CTX + MMF
7	F	3.6	2	Hypertension, weak pulse, finger ulcer, malaise	5/5/8	15	R	GC
8	F	3.3	5	Malaise, fever, anorexia	2/2/5	16	R	IVIG + GC
9	F	4.1	2	Malaise, anorexia	0/1/4	13	R	IVIG + GC + CTX + MMF
10	F	3.5	22	Vomit, hypertension, fever, malaise	3/3/6	-	D	GC

Mo, month; D, day; PVAS, pediatric vasculitis activity score; ITAS, Indian Takayasu Clinical Activity Score; R, remission on treatment; D, deceased; IVIG, intravenous immunoglobulin; GC, glucocorticoid; CTX, cyclophosphamide; MMF, mycophenolate mofetil; IFX, infliximab; TCZ, tocilizumab; COX, cyclosporine.

### 3.3 Laboratory and imaging results

At the time of presentation, all infants included had elevated CRP, ESR, and white blood cell (WBC) counts and low hemoglobin levels. At the same time, high levels of brain natriuretic peptide (BNP) (55.6%) and ferritin (40%) are also shown in Table 2. In addition, two of the three patients who underwent genetic sequencing were found to have abnormal genetic deoxyribonucleic acid (DNA) sequences, including VWF mutation in patient 3 and CLCN5 mutation in patient 10, which could not explain the occurrence of vasculitis (7, 8).

**Table 2:** Laboratory text results and echocardiography results.

Examination	Values	Abnormal number	Proportion (%)
CRP (mg/L)	99.93 ± 64.30	10/10	100
ESR (mm/h)	76.1 ± 31.5	10/10	100
WBC count (10^9^/L)	18.62 ± 4.70	10/10	100
Hemoglobin (g/L)	89.3 ± 12.5	10/10	100
Platelet count (10^9^/L)	527.9 ± 302.2	7/10	70
BNP (pg/ml)	432.0 ± 508.4	5/9	55.6
Ferritin (ng/ml)	376.0 ± 363.5	4/10	40
Autoantibodies	/	2/7	28.6
Gene sequence	/	2/3	66.7
Echocardiography	/	7/10	70

CRP, C-reactive protein; ESR, erythrocyte sedimentation rate; WBC, white blood cell; BNP, brain natriuretic peptide.

Computed tomographic angiography (CTA) was performed in nine patients at diagnosis, which revealed thickening of the arterial wall (9/9), long stenosis (9/9), vessel dilatation (5/9), aneurysms (3/9), and reduced blood flow (1/9). magnetic resonance angiography (MRA) of patient 2 showed lumen narrowing, vessel dilatation and coronary artery aneurysm.

In all patients, the most frequently affected arteries were the thoracic aorta (six infants, 60%) and abdominal aorta (six infants, 60%), followed by the aortic arch (four infants, 40%), renal artery (four infants, 40%) and coronary artery (four infants, 40%). The details are presented in Table 3.

**Table 3:** Involved vessels.

Involved vessel	Number	Proportion (%)	Involved vessel	Number	Proportion (%)
Ascending aorta	2	20	Abdominal aorta and its branches	1	10
Aortic arch and its branches:	2	20	Abdominal aorta	6	60
Aortic arch	4	40	Celiac trunk	3	30
Coronary artery	4	40	Superior mesenteric artery	3	30
Thoracic aorta	6	60	Inferior mesenteric artery	2	20
Renal artery	4	40	Iliac artery	2	20
			Pulmonary artery	2	20

Based on the angiographic classification of TAK (International Conference of Takayasu's arteritis in Tokyo, 1994) (4), the most common angiographic involvement type was type III (three infants; 30%) and type V (three infants; 30%), followed by type IV (two infants; 20%), type IIa and type IIb in one infant each. None showed type I involvement (Table 4). In addition, positive echocardiography results were observed in 7 subjects (70%).

**Table 4:** Distribution of involved vessels according to the new angiographic classification of Takayasu arteritis.

Type	Types of affected vessels	Numbers of patients (*N* = 10)
I	Branches from aortic arch only	0
IIa	Ascending aorta, aortic arch and its branches	1 P+
IIb	Ascending aorta, aortic arch and its branches, thoracic descending aorta	1
III	Thoracic descending aorta, abdominal aorta and/or renal arteries	3 (2/3C+)
IV	Abdominal aorta and/or renal arteries	2 (1/2C+; 1/2C + P+)
V	Combined features of types IIb and IV	3 (1/3C+)

According to this classification system, involvement of the coronary or pulmonary arteries is designated as C(+) or P(+), respectively.

### 3.4 Disease activity scores and correlations between disease activity indexes

**Figure 1:**
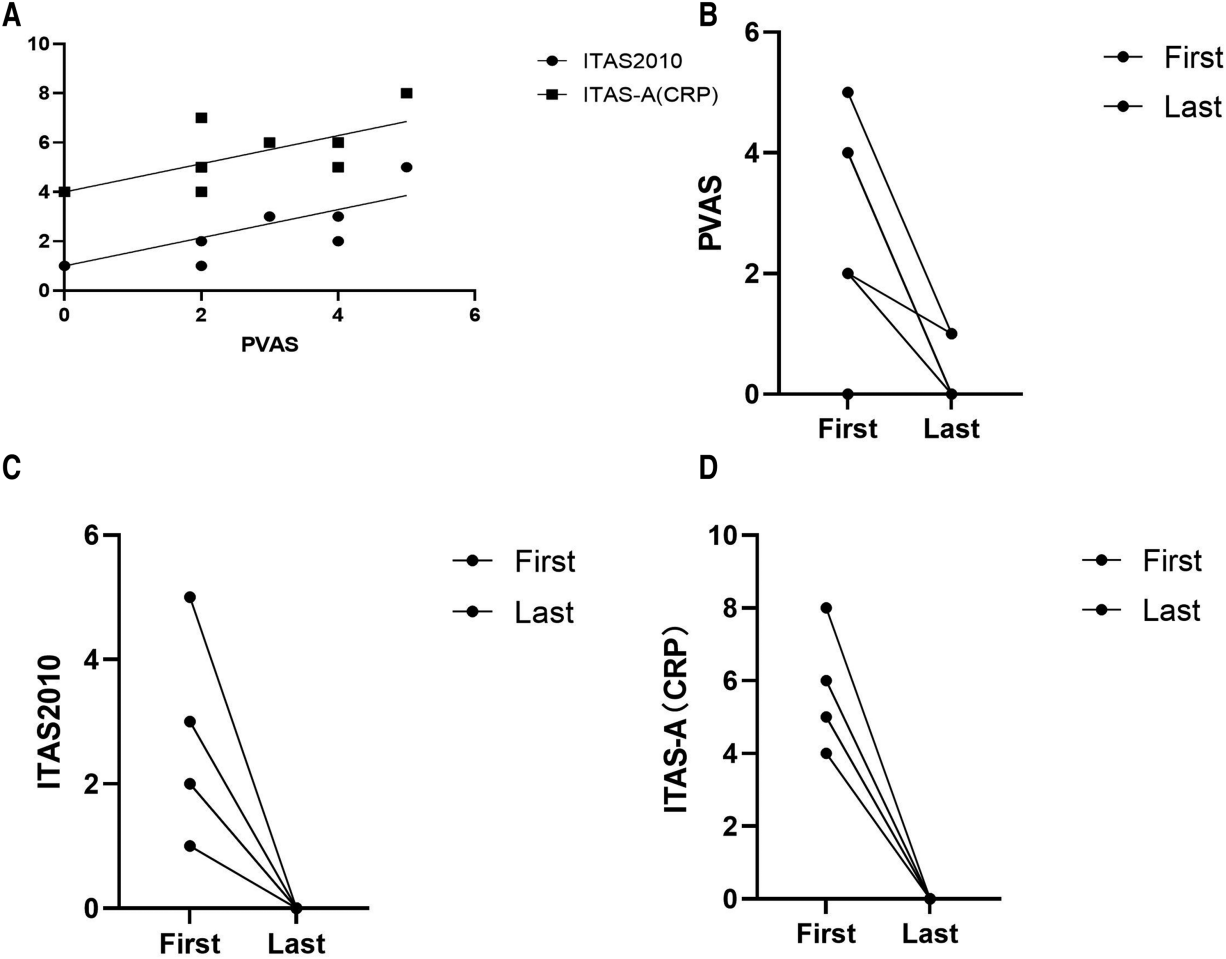
(**A**) Correlation between takayasu arteritis disease activity measures at the time of diagnosis: ITAS-2010, *r*^1^ = 0.444, *P* = 0.035; ITAS-A, *r*^2^ = 0.444, *P* = 0.035. (**B–D**) PVAS, ITAS2010 and ITAS-A at the first clinic visit and at the last follow-up, respectively. ITAS2010 (*p* = 0.001) and ITAS-A (*p* = 0.000) decreased significantly among the eight patients alive.

At the first visit to the clinic, the mean PVAS was 2.8/63 (range, 0–5); ITAS was 2.6/51 (range, 1–5); ITAS-A was 5.6/54 (range, 4–8). The disease activity scores are presented in Table 1. The PVAS showed a strong correlation with ITAS (*r* = 0.444; *P* = 0.035) and ITAS-A (*r* = 0.444; *P* = 0.035) (Figure 1A) at the first clinic.

### 3.5 Treatment and outcomes

All 10 infants were treated with GCs (1–2 mg/kg). Three of the infants were treated with GCs alone, one of whom died from severe infection, while the others achieved the remission of symptoms and inflammation indexes quickly. The other seven infants were treated with GCs combined with intravenous CYC monthly for three to six months. After three CYC treatments, case 2 switched to anti-interleukin 6 receptor antibody (tocilizumab, TCZ) (12 mg/kg, monthly), while case 4 deceased from aneurysm disruption. After combination therapy with GCs and CYC for 6 months in the remaining five infants, MMF combined with a low dose of oral GCs was initiated in four cases, except that oral GCs alone were used in case 5. Prior to the treatment of GC alone or GC with CYC, intravenous immunoglobulin (IVIG) (2 g/kg, once or twice) was used in seven infants, infliximab (IFX) in four infants and cyclosporine (COX) in 1 infant. However, their temperature and inflammation indexes did not decrease, indicating the failure of therapy (Table 1).

**Figure 2:**
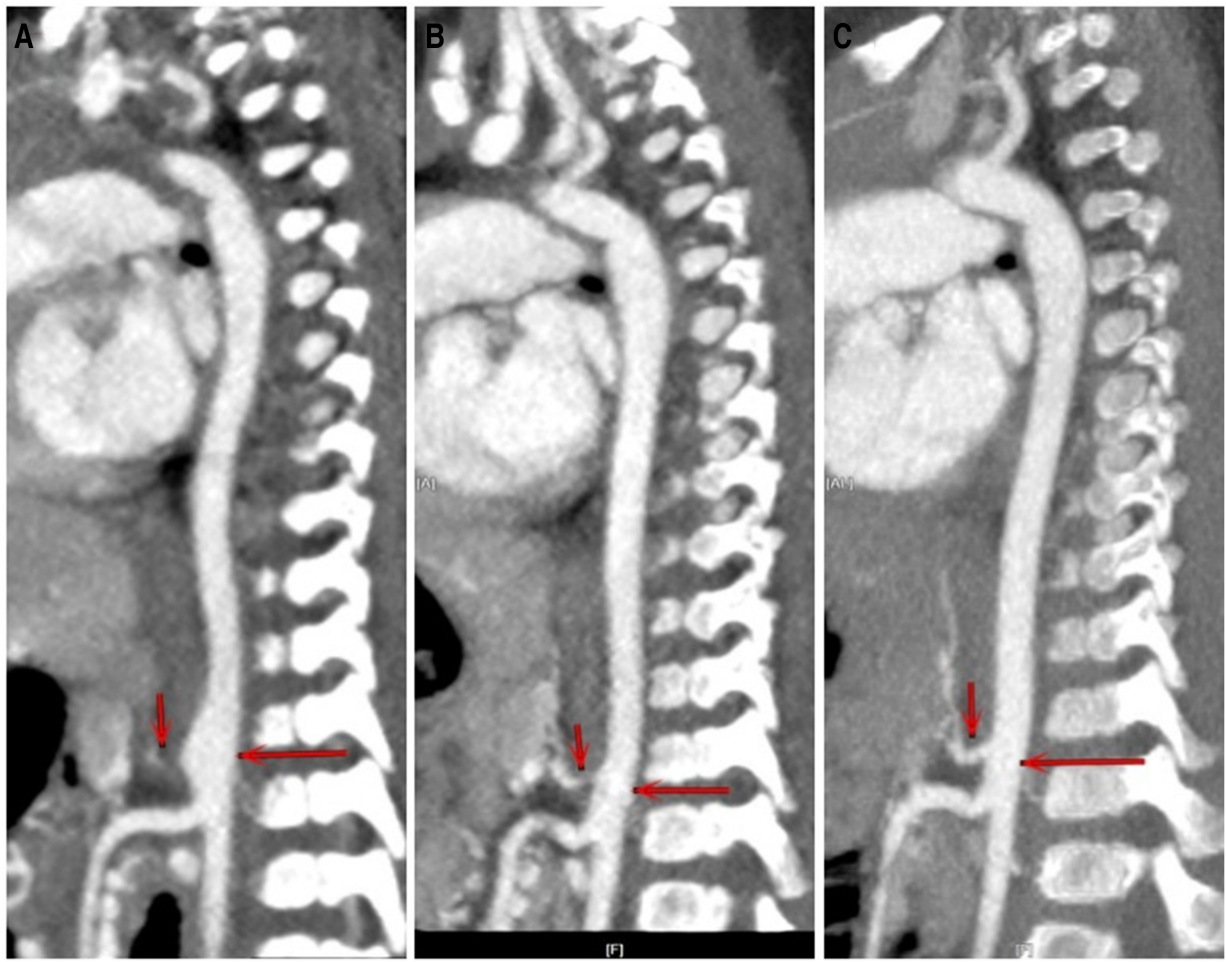
CTA of case 1 at different stages. (**A**) At first clinic visit. (**B**) After three and a half months of treatment. (**C**) After 1 year of treatment. The involved aorta improved to a great extent after treatment.

With continued treatment, clinical manifestations disappeared, inflammation indexes decreased to the normal range, the involved vessels improved to a great extent, and the disease activity scores decreased significantly among these eight patients (Figures 1B–D, 2).

## 4 Discussion

TAK is a systemic vasculitis primarily involving the aorta and its main branches, showing a predominance in child-bearing women. Although rare, it does occur in childhood, even in infancy (9). Due to the lack of salient clinical features and specific biomarkers, diagnosis remains challenging, resulting in a delay in diagnosis, which leads to a poor prognosis in TAK (10). In the present retrospective study, we reported a cohort of infantile patients with TAK who were rarely reported, making our results meaningful and useful for clinicians and researchers.

To date, the candidate genes implicated in pathogenesis include human leukocyte antigen (HLA)-B loci (HLA-B52), IL-12B, IL-17F, and CRP genes, making the host genetically susceptible (11–13). In our study, abnormal genetic DNA sequences, including VWF and CLCX5, were found in patient 3 and patient 10, respectively. VWF plays an essential role in regulating the balance between blood clotting and bleeding, and the CLCX5 gene is related to Dent disease, while the correlation between both genetic mutations and the occurrence of disease has not been found in the literature (7, 8). Therefore, further studies are needed to identify alleles responsible for susceptibility in this early-onset group. However, genetic analysis is still necessary in this early-onset group since TAK may co-exist with immunodeficiencies as described (14).

In contrast to other case series, the most common presenting manifestation of TAK in our study was malaise (80%), followed by fever, rather than the hypertension reported previously (15, 16), which was not specific at all to TAK, partly explaining the delay in diagnosis. In addition, we emphasized that in infants, it is of great significance and difficult to distinguish TAK from other diseases since a plethora of conditions can mimic TAK (17). For example, Kawasaki disease (KD), another vasculitis predominantly affecting infants and children younger than 5 years old, manifests as persistent fever and characteristic changes in the skin, mucous membrane, and lymph nodes with elevated CRP and ESR (18), which may also present in TAK patients. In our cohort, more than half of infants were initially misdiagnosed with KD, leading to poor results after remedies targeting KD. Thus, TAK should be considered when febrile infants do not respond to antibiotic therapy with nonspecific systemic symptoms but elevated levels of acute phase reactants (CRP and ESR) in addition to KD. Moreover, TAK should also be noted when a KD infant is resistant to medications for KD, especially in incomplete KD. On the other hand, multiple reports have proven that KD does not affect coronaries alone: large vessels can be involved as well, leading to systemic artery aneurysms (SAA) (19, 20), making it more challenging to differentiate it from TAK. In this condition, systemic assessments including involvement of aorta, change of platelet count, response to IVIG and self-limiting course may aid in the differential diagnosis of these two diseases. Moreover, patients diagnosed with KD complicated with SAA should firstly meet the clinical criteria for the diagnosis of KD. In addition, at the early stage, wall thickness and luminal narrowing are more common in TAK patients while dilatation of coronary arteries is observed commonly in KD children.

Of note, the median time to diagnosis in our cohort was 9.5 days, significantly shorter than that reported previously (16, 21), which may be attributed to the clinicians' elevated awareness of very early-onset TAK in recent years in our center. In addition, we described a patient with finger ulcer, which, to our knowledge, has never been reported in I-TA.

Similarly, in this study of 10 patients from our center who had disease onset of TAK before the age of 3 years, we also observed elevated CRP and ESR levels in all infants, both of which are closely correlated with disease activity. Moreover, compared with reports in childhood-onset TA (c-TA) and adulthood-onset TA (a-TA), the rate of elevated acute phase reactant levels was obviously higher in I-TA, which may demonstrate the existence of overactive inflammation and a higher risk of thrombotic events since the association of increased CRP with thrombosis has been verified (22). A number of researchers are searching for novel circulating biomarkers specific to TAK that are useful for the assessment of disease activity, including matrix metalloproteinase-2 (MMP-2), MMP-3, MMP-9, interleukin-6 (IL-6), pentraxin (PTX3), amyloid A and so on (23). Among these indexes, PTX-3 and serum amyloid A were reported to be convincing in TAK patients according to multiple related studies (24–26). However, multicenter and prolonged studies are needed to confirm these newer assays prior to wide application in clinical practice.

It is well established that imaging plays an essential role in assessing disease activity and extent. Similar to previous findings (15, 16), we also found that the most common type of vessel involvement was type V, and the most commonly involved vessels were the abdominal aorta and thoracic aorta. However, in contrast to c-TA, we found that vascular involvement is highly variable in these I-TAs, which predicts a worse prognosis, requiring strengthened therapy as soon as possible to control inflammation and avoid further organ damage. It is noteworthy that cardiac ultrasound provided significant evidence for diagnosis in our study, showing dilation of the coronary artery or pulmonary artery, aneurysm of the coronary artery, auxo-cardia and abnormal blood flow, which indicates that TAK can also affect medium-sized arteries even as well as large-vessel vasculitis.

To date, no recognized standard has been established to evaluate TAK disease activity, although multiple tools have been used. ITAS2010/ITAS-A is a special scale for TAK that has been validated in adults, while PVAS is a special scale for vasculitis activity in children (5, 6). We explored the activity of ITAS2010/ITAS-A in comparison with PVAS, finding unsurprisingly that there was a good correlation between PVAS and ITAS2010/ITAS-A at diagnosis. However, it is reported that I-TA has a more severe disease than c-TA (27) suggesting that the development of a new and specific tool for I-TA disease activity is warranted taking the age of disease onset into account.

GC is the mainstay treatment for TAK, combined with immunosuppressive therapy or biological agents, especially for glucocorticoid-resistant or glucocorticoid-refractory patients (28). For our infants, GCs combined with immunosuppressive agents, which included CYC used as an induction drug for 6 months and MMF used as a maintenance drug, were the standard therapy with good results, except for one patient who died from acute aortic rupture. Likewise, a prospective study in a-TA revealed that the application of CYC is beneficial to reduce the unfavorable outcomes of TAK (29). In accordance with the experience from a tertiary center in South India, MMF has been used consistently in both a-TA and c-TA with satisfactory outcomes (30). Another meta-analysis also confirmed the efficacy of MMF for the control of disease activity and to taper the GC dosage (31). Other than these conventional immunosuppressants, biologic agents (mainly including IFX and TCZ) have also been attempted given their superiority in the control of inflammation and the reduction in acquired GC dose (32, 33). To date, promising results have been reported with TCZ for c-TA, particularly for refractory patients (33). In our study, only one patient switched from CYC to TCZ combined with GCs for worrying about the adverse complications of CYC. Additionally, the benefits of tumor necrosis factor (TNF) inhibitors have been verified by many reports (34, 35). However, in our cohort, four infants (cases 2, 4, 5, and 6) were prescribed IFX once misdiagnosed with IVIG-resistant KD, yet no total remission was observed, manifesting as recurrence or persistence of fever, abnormal levels of inflammation indexes and/or extensive involvement or no improvement in vascular imaging, which urged us to adjust the treatment protocol to classic GCs combined with CYC. Other kinds of biologics, such as ustekinumab (UST) and rituximab, have also been tried with success in TAK (32, 36). However, limited data cannot draw convincing conclusions regarding these biologics as an option for treatment in I-TA. Of course, surgical intervention is recommended if necessary (2, 33).

Interestingly, IVIG, even repeated usage, did not work in controlling inflammation at all in I-TA in our study, which is in disagreement with a previous case report from Poland (37).

The mortality rate of our patients was 40%, which was much higher than that reported in a-IA and c-TA (15, 38). In our study, case 4 succumbed to aneurysm disruption owing to the inflammation of vessels, which was not controlled completely. Another infant dying from severe infection reminds us to pay attention to the complications secondary to the medications and procedures as well as TAK-related complications.

Several limitations should be addressed. First, only 10 infants were enrolled in our case series, and the follow-up time was relatively short. Second, the retrospective design is also a limitation of our study. Third, there is a risk of selection bias since it was not a population-based study. Finally, our single-center data might not reflect the whole spectrum of I-TA in China.

In conclusion, TAK in infants is rare but potentially life-threatening. Nonspecific systemic symptoms and a lack of biomarkers make it challenging to establish an early diagnosis. We should pay more attention to I-TA since it can involve more vessels, cause more severe inflammation and lead to higher mortality. A new tool to assess disease activity in I-TA may be needed. Although the treatment of TAK in infants has not been clearly documented, it is of great importance to treat TAK aggressively as soon as the diagnosis is secured to improve prognosis. In I-TA, the use of GCs and CYC may control disease activity.

## Data Availability

The raw data supporting the conclusions of this article will be made available by the authors, without undue reservation.
